# Ketogenic diet enhances the anti-cancer effects of PD-L1 blockade in renal cell carcinoma

**DOI:** 10.3389/fendo.2024.1344891

**Published:** 2024-05-17

**Authors:** Jeremy Richard, Céline Beauvillain, Maxime Benoit, Magalie Barth, Cécile Aubert, Cyrielle Rolley, Sarah Bellal, Jennifer Bourreau, Matthieu Ferragu, Souhil Lebdai, Arnaud Chevrollier, Daniel Henrion, Vincent Procaccio, Pierre Bigot

**Affiliations:** ^1^ MITOVASC, SFR ICAT, Univ Angers, CHU Angers, Inserm, CNRS, Angers, France; ^2^ Univ Angers, Nantes Université, CHU Angers, Inserm, CNRS, CRCI2NA, SFR ICAT, Angers, France; ^3^ Urology Department, Angers University Hospital, Angers, France; ^4^ Departement de Pédiatrie, CHU d’Angers, Angers, France; ^5^ Department of Pathology, Angers University Hospital, Angers, France

**Keywords:** renal cell carcinoma, metabolic reprogramming, mitochondrial biogenesis, PDL1, adjuvant ketogenic diet, immunotherapy

## Abstract

**Introduction:**

Clear cell renal cell carcinoma (ccRCC) is characterized by a predominant metabolic reprogramming triggering energy production by anaerobic glycolysis at the expense of oxydative phosphorylation. Ketogenic diet (KD), which consists of high fat and low carbohydrate intake, could bring required energy substrates to healthy cells while depriving tumor cells of glucose. Our objective was to evaluate the effect of KD on renal cancer cell tumor metabolism and growth proliferation.

**Methods:**

Growth cell proliferation and mitochondrial metabolism of ACHN and Renca renal carcinoma cells were evaluated under ketone bodies (KB) exposure. *In vivo* studies were performed with mice (nude or Balb/c) receiving a xenograft of ACHN cells or Renca cells, respectively, and were then split into 2 feeding groups, fed either with standard diet or a 2:1 KD ad libitum. To test the effect of KD associated to immunotherapy, Balb/c mice were treated with anti-PDL1 mAb. Tumor growth was monitored.

**Results:**

*In vitro*, KB exposure was associated with a significant reduction of ACHN and Renca cell proliferation and viability, while increasing mitochondrial metabolism. In mice, KD was associated with tumor growth reduction and PDL-1 gene expression up-regulation. In Balb/c mice adjuvant KD was associated to a better response to anti-PDL-1 mAb treatment.

**Conclusion:**

KB reduced the renal tumor cell growth proliferation and improved mitochondrial respiration and biogenesis. KD also slowed down tumor growth of ACHN and Renca *in vivo*. We observed that PDL-1 was significantly overexpressed in tumor in mice under KD. Response to anti-PDL-1 mAb was improved in mice under KD. Further studies are needed to confirm the therapeutic benefit of adjuvant KD combined with immunotherapy in patients with kidney cancer.

## Introduction

Renal carcinoma is the 6th cause of cancer’s death in industrialized countries but also the third cancer of the urogenital tract. The incidence of renal cancer in Europe reach almost 115 000 new cancers per year (1, 2). At diagnosis, 10 to 20% of renal cell carcinoma (RCC) patients have synchronous metastases and 10 to 30% will develop metachronous metastases after nephrectomy for localized disease. Landmark advances has been made over the last years in frontline treatment of metastatic RCC with the arrival of immune checkpoint inhibitors (ICI) and tyrosine kinase inhibitors based combinations (3–5). The recommended treatment is currently a combination of pembrolizumab plus axitinib, nivolumab plus ipilimumab, nivolumab plus cabozantinib or pembrolizumab plus lenvatinib for RCC (6). Overexpression of ICI targets such as PD-L1 on the surface of tumor cells is considered as a positive marker of response to metastatic renal cancer treatment (7).

Around the 1920s, O. Warburg described a metabolic switch in tumors, characterized by an energy production through glycolysis and no longer by oxidative phosphorylation (OXPHOS) (8). The aerobic glycolysis enhanced tumoral proliferation (9), tumoral invasion and disease progression (10). A study, based on the Cancer Genome Atlas Research Network confirmed the important role of the « metabolic switch » in renal carcinogenesis (11). Moreover, Courtney and al. demonstrated by using infused [U-13C] glucose into patients with primary Renal Cell Carcinoma (RCC) tumors undergoing nephrectomy, enhanced glycolysis and a suppression of glucose oxidation in tumors compared to matched adjacent non tumoral kidney tissue. It was the most important demonstration that primary RCC tumors had a distinctive metabolic profile, providing strong *in vivo* evidence for the Warburg effect in human renal tumors (12).

The ketogenic diet (KD) is a diet rich in fats with reduced glucose concentration. This diet forces the body to produce energy by ketolysis rather than by glycolysis (13, 14). Recently, the efficacy of KD was proven slowing down tumor growth and increasing the survival of animals with malignant glioma (15), neuroblastoma (16), gastric cancer (17) and prostate cancer (18, 19). The hypothesis is that normal cells could keep an efficient energetic metabolism, due to ketone bodies (lipid intake) and OXPHOS. The tumor cells, which under-express the respiratory chain proteins, could not metabolize the ketogenic bodies for their energetic production. Low glucose intake could favor the Krebs cycle through Acetyl-CoA and fatty acid beta-oxidation, limiting glycolysis and depriving the cancer cells of their principal energetic substrate. Previous studies demonstrated the feasibility and the excellent tolerance of KD to treat human cancers, without inducing any serious adverse events, and especially no alteration of the lipid profile (20, 21). Here, we analyzed *in vitro* models and preclinical tumor mouse models to better understand the functional impact of KD on renal cancer cell proliferation and mitochondrial metabolism.

## Materials and methods

### Patients

Eighteen patients with kidney tumor were included in this study. Kidney specimens were obtained. These patients were treated by nephrectomy for renal cancer in our center between January and May 2021. We were able to establish primary cultures of clear cell renal cancer in 8 of them. The clinical and pathological data of those 8 patients are reported in **Supplementary Table 1** of the **Supplementary Data**.

### Primary cell isolation and culture

Once the nephrectomy was performed, the surgical specimen was transported in a fresh buffered salt solution to the department of Pathology for macroscopic examination. After anatomopathological analysis, a 1 cm^3^ non-necrotic fragment of the tumor was selected and stored at 4°C in 50 mL of PBS (phosphate-buffered saline, Sigma Aldrich, St. Louis, MO, USA) until use. Following this, mechanical digestion was performed under sterile conditions using two cold scalpels. The resulting millimeter-sized pieces were incubated at 37°C for 90 min under agitation in the presence of DMEM 25mM glucose, 1mM pyruvate, 1% penicillin–streptomycin (Sigma Aldrich), 0.2 Wünsch units/mL liberase (Roche, Basel, Switzerland) and 0.1 mg/mL DNase (Roche). The solution obtained was sieved through a 70 μm filter (SPL Life Sciences, Pocheon, Republic of Korea). Suspended cells were pelleted for 5 min at 300× g. Next, cells were subcultured in culture medium 1 containing 33% of Mix AmnioMAX™ (AmnioMAX™medium + AmnioMAX™ supplement) (Thermo Fisher Scientific, Waltham, MA, USA), 10% of Fetal Bovine Serum (FBS) (Thermo Fisher Scientific), 1% of RGEM1™ (LONZA, Basel, Switzerland), 2mM of Glutamine (Sigma-Aldrich), 1% of penicillin and streptomycin (Sigma-Aldrich) and 54% of DMEM 25mM of glucose (Sigma-Aldrich) prewarmed to 37°C and transferred to 25 cm2 culture flasks (SPL Life Sciences). After overnight incubation (37°C, 5% CO2), culture medium was renewed, and the unattached cells were removed. This procedure was repeated every 24 h until the flasks were free of debris and unattached cells. Thereafter, the culture medium was changed every 2 to 3 days until confluence. At 70–80% confluence, cells were washed with PBS and detached using trypsin (20 μL/cm2 trypsin-EDTA 1X (trypsin 0.05%; EDTA 0.02%), Sigma-Aldrich) for 5 min. Cells were then pelleted by 5 min centrifugation at 500× g and resuspended in 2 mL of culture medium 2 containing 33% of Mix AmnioMAX™ (Thermo Fisher Scientific), 10% of FBS (Thermo Fisher Scientific), 2mM of Glutamine (Sigma-Aldrich), 56% of DMEM 25mM of glucose (Sigma-Aldrich). Cell count was performed by trypan blue exclusion and Neubauer hemocytometer. Some of the cells were frozen at −80°C, and the rest were transferred to a 75 cm2 flask with culture medium 2. Once 80% confluence was obtained, the cells were transferred to a T175 cm2 flask for amplification. Culture medium was optimized for survival after mechanical and chemical digestion and to avoid culture infections during tissue handling. It contained growth factor supplements for use with renal epithelial cell basal medium (1% REGM™ (Renal Epithelial Cell Growth Medium SingleQuots™ Kit, LONZA, Bâle, Switzerland)) and 1% antibiotic (penicillin and streptomycin, Sigma-Aldrich). After the first passage, antibiotics were no longer needed and could constitute an avoidable bias for cell growth.

### Cell culture

The ACHN cells (kindly provided by the laboratory of translational genomics at the National Cancer Institute) and Renca cells were cultured at 37C° in a humidified atmosphere (95% air; 5% CO2) in standard DMEM high glucose media (25mM) (PAN biotech, Aidenbach, Germany), supplemented with 10% Fetal Bovine Serum (FBS) and 2mM glutamine (PAN biotech). The medium was changed every 2 days. The doses of low glucose 2.8mM (LG) and ketone bodies (KB) were chosen in agreement with concentrations used in previous *in vitro* studies (22). For the KB treatment, 5 mM of acetoacetate and β-D-hydroxybutyrate (Sigma Aldrich, Lyon, France) were added to the low glucose media (2.8mM) supplemented with 10% SVF and 2mM glutamine, and cells were collected after 6 days of culture for further analysis.

### Measurements of cell growth proliferation and viability

To assess cell viability in response to glucose starvation and KB exposure, we used an automated live cell imaging (IncuCyte ZOOM system, Sartorius) in which cell density and shape were observed by photonic microscopy while cells were maintained under standard medium or exposed to KB as described above. Real-time live-cell images were taken every 2 hours and for analysis of cell viability, well confluence (%) was automatically calculated during 144h by using the Basic Analyzer segmentation mask (IncuCyte ZOOM software 2015A). Cell viability was analyzed by Crystal Violet staining as described elsewhere (23). Absorbance was measured using a microplate reader at 590 nm (CLARIOstar®, BMG Labtech).

### Mitochondrial enzymatic activities

Citrate synthase activity, considered as a marker of mitochondrial mass was measured by adding cells to a reaction mix (DTNB 1mM, oxaloacetic acid 10 mM, acetyl coA 0.3 mM, triton X100 0.1%) and the appearing rate of CoA-SH was measured at 37°C at 412nm on an UVmc2 spectrophotometer (SAFAS, Monaco) as described elsewhere (24).

### Mitochondrial respiration rate measurements

Mitochondrial oxygen consumption measurements were performed at 37°C and atmospheric pressure using a high-resolution oxygraph (O2K, Oroboros instrument, Innsbruck, Austria). Respiration rates on intact cells (4x10^6^ cells) were measured in standard medium or low glucose with added ketone bodies (KB) as described elsewhere (24). Oxygen consumption rate (OCR) and Extracellular acidification rate (ECAR) were also measured with an XF96 extracellular flux analyzer (Agilent Technologies, Santa Clara, CA) to determine the bioenergetic profile of intact cells (30 × 10^3^ cells/well) after being exposed to KB or HG as recommended by the manufacturer. Three independent replicates of each oxygen consumption measurement were generated, and results were normalized according to cell concentration.

### Metabolites and NADH/NAD^+^ measurements

Glucose, pyruvate and lactate concentrations were measured by spectrophotometry with a Xenius apparatus (SAFAS, Monaco) according to the manufacturer (Abcam, Paris France). The cytosolic NAD(H)-redox state was determined using the metabolite indicator method, based on the lactate dehydrogenase reaction, by measuring pyruvate to lactate ratio in cell culture.

### MtDNA copy number quantification

To determine the mtDNA copy number in human cells, quantitative PCR (qPCR) was performed by SYBR Green incorporation using the Chromo4 System (Biorad) following the manufacturer recommendations. Selected primers were respectively, MT-ND4 (Forward: actctcactgcccaagaact and Reverse: gtgtgaggcgtattatacca) and MT-COX1 (Forward: tacgttgtagcccacttccact and Reverse: agtaacgtcggggcattccg) to quantify the mtDNA copy number, and GAPDH (Forward: ccctgtccagttaatttc and Reverse:caccctttagggagaaaaa) and AIBI (Forward: ggagtttcctggacaaatga) and Reverse:aggactggcgtttatgtctt) to quantify the nuclear DNA in ACHN cells. The PCR reactions were performed as follows: initial denaturing at 95°C for 10 min, and 35 cycles at 95°C for 30 s, 60°C for 1 min. A melting curve was analyzed in order to check the specificity of the PCR products.

### RNA extraction and quantitative PCR measurements

Total RNA was extracted using RNeasy Micro kit following the recommendations of the manufacturer (Qiagen, Hilden, Germany). A Bioanalyzer 2100, with a RNA6000 Nano kit (Agilent Technologies, Santa Clara, CA, U.S.A.) was used to assess RNA quality. The RNAs was reverse transcribed using Superscript II reverse transcriptase (Invitrogen, Waltham, Ma). Expression of selected genes including Nuclear Respiratory Factor 1 (*NRF1)*, Nuclear Respiratory factor 2 (*NRF2)*, Peroxisome Proliferator-Activated Receptor Gamma Coactivator 1-Alpha (*PGC1α)* and Transcription factor A (*TFAM*) and PDL-1 or CD274 was analyzed by SYBR Green fluorescent dye incorporation using the Chromo4 System (Biorad) as described elsewhere (25). Specific gene expression was calculated using the 2^-ΔΔCT^ method using Actin B for normalization. Primer sequences are available upon request.

### Animal studies

Twenty females (two experiments, 5 mice per group and per cage) eight-week-old CD-1 nude mice (Charles River’s laboratory, France) were maintained in a temperature-controlled room with food and water provided ad libitum. 2 × 10^6^ ACHN cells were subcutaneously grafted in the flank of nude mice. Mice were randomized into two groups, either fed with a standard diet (control group) or with a 2:1 high fat ketogenic diet (ketogenic group).

Standard diet (*ssniff Spezialdiäten GmbH, Soest, Germany)* contained 16.1% proteins, 7.1% lipids and 57.2% glucids, resulting in a 0.09:1 ketogenic ratio. KD, TCM completed (*ssniff Spezialdiäten GmbH, Soest, Germany*) contained 13.8% proteins, 56.1% lipids (31.1% TCL, 25% TCM-C10) and 12.5% glucids, resulting in a 2.1:1 ketogenic KD diet was provided by the Vitaflo company (*Vitaflo International Ltd, Liverpool, New England).* Tumor progression was monitored weekly by ultrasounds for 8 weeks *(Vevo 770^®^*, *VisualSonics, Toronto, Canada*). Tumor volume was calculated using the empirical ellipsoid formula: V (mm3) = *4/3 π x (l/2) x (w/2) x (d/2)*, with l for length, w for width and d for depth. The tumor growth (growth percentage) was calculated according to the (V - Vi)/Vi formula, V standing for the measured volume and Vi for the initial volume. Glycemic blood and blood ketone levels were monitored by standard devices (*Freestyle Optium Neo, Abbott Laboratories, Illinois, USA*) after tail section, twice a week. Eight weeks after xenografts, mice were sacrificed by cervical dislocation after isoflurane anesthesia. Tumors were removed from animals, measured and weighed, then quickly frozen and stored at -80°C.

For the second assay, twenty 7–8-week-old female BALB/c mice (Janvier Labs, Le Genest Saint Isle, France) (two experiments, 5 mice per group and per cage) were inoculated with 5×10^5^ Renca tumor cells (ATCC, CRL-2947) subcutaneously at day 0 and then injected with the indicated treatment. Isotype control (LTF‐2 clone) and anti-PDL1 antibody (10F.9G2 clone, Bio-XCell, West Lebanon, NH, USA) or vehicle (PBS) were conducted by intraperitoneal injection (200µg/mouse) every 3 days. Ketogenic or standard diet was administered ad libitum. Tumor volumes were measured and calculated with the equation: volume=length×width^2^/2. Survival was performed using human endpoints, mice were sacrificed when an endpoint was reached to comply with ethical regulations. This means that experiments were terminated when mice reached defined criteria (tumor size length>17mm; weight loss > 20%, or ulceration at the tumor site). Twelve days after tumoral cell injection, animals were pooled and randomly divided into 2 designated experimental groups one with ketogenic diet and the second with standard diet. Each group was subdivided in 3 groups, one received anti-PDL1 mAb, the second isotype control and the third PBS (vehicle).

### Immunohistological studies of murine tumors

All tissue samples were formalin-fixed, paraffin-embedded and cut into 4-μm-thick sections. Slides were stained with hematoxylin, eosin and saffron (HES). Immunohistochemical staining was carried out with rabbit monoclonal anti-PD-L1 antibody (clone NAT-105, Leica,1:200 dilution, Germany) and anti-mib1 antibody (clone ab15580, Abcam, 1:500 dilution, UK), on a Bond-III automated immune stainer (Leica Microsystem, Nussloch, Germany). After heat antigen retrieval during 20 min in ER2 pH 9 buffer, sections were incubated at room temperature for 20 min with the primary mouse monoclonal anti-PDL-1 antibody (ab205921, abcam) at a 1:500 dilution and were revealed with the Leica Bond Refine detection kit for 10 min. The specimens were then counterstained with hematoxylin (both from Leica Microsystems, Newcastle, U.K).

### Statistical analysis

Statistical analysis was conducted using Graphpad software (Prism version 8.0.1). Differences between groups were evaluated by the Mann-Whitney statistical test. The asterisk (*, ** and ***) indicates significant differences (respectively *p* < 0.05, *p* < 0.01 and *p* < 0.0001). Analysis of tumor growth between the two experimental groups was performed using the Student’s t test. Kaplan-Meier curves and corresponding Gehan-Breslow-Wilcoxon tests were used to evaluate the statistical differences between groups for the survival studies. A p-value <0.05 was considered to be statistically significant.

## Results

### Ketogenic diet reduced *in vitro* growth proliferation of renal cancer cells with a switch towards oxidative metabolism

**Figure 1:**
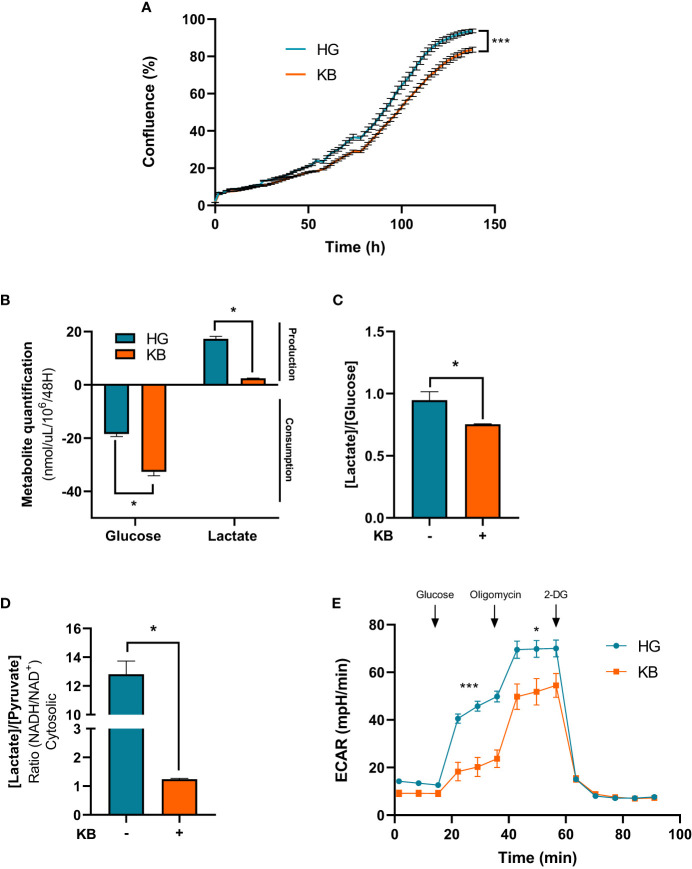
Ketone bodies reduced growth cell proliferation and increased mitochondrial respiration and biogenesis of ACHN cells. HG = 25mM glucose, 2mM L-glutamine, 10% non-dialyzed FBS, 0mM ßHB, 0mM AcAc and KB = 2.8mM glucose, 2mM L-glutamine, 10% non-dialyzed FBS, 5mM ßHB, 5mM AcAc **(A)** Quantification of ACHN cell growth exposed to KB compared to untreated cells (HG)for 6 days was assessed by Incucyte. **(B)** Glucose consumption and lactate production were assessed in cell supernatant from untreated HG-ACHN compared to KB-ACHN (n=4). **(C)** Ratio of lactate/glucose was estimated in supernatants from HG-ACHN and KB-ACHN (n=4). **(D)** Cytosolic NADH/NAD+ ratio was determined from the pyruvate/Lactate concentration ratio (n=4). Data are presented as mean ± SEM. Asterisks (*) indicate significant differences (p<0.05), (**) (p<0.001), (***) (p<0.0001). **(E)** ECAR production measurements (n=6).

The ACHN cells were cultured in low glucose medium enriched in KB or standard medium during 6 days. At day 6, growth proliferation of ACHN cells was significantly reduced by 11% in the presence of low glucose in addition to ketone bodies compared to HG medium (**Figure 1A**). Similar results of growth proliferation were reduced in LG with added KB treated primary kidney cancer cells (**Supplementary Figure**). To better understand the reduction of growth cell proliferation under LG with added KB, metabolites in cell culture supernatants were quantified. Glucose consumption was significantly increased by 77% in ACHN cells under low glucose in addition to ketone bodies media, also associated with a significant reduction of 86% of lactate production (**Figure 1B**). The reduction of lactate was confirmed with 24% decrease of the [lactate]/[glucose] ratio (**Figure 1C**). The cytosolic NADH/NAD+ ratio was estimated by using the lactate/pyruvate (L/P) ratio at equilibrium and was significantly reduced (90%) for ACHN cells exposed to low glucose coupled with ketone bodies compared to high glucose medium, suggesting a switch from glycolysis to oxidative metabolism (**Figure 1D**). The level of ECAR of ACHN cells was significantly reduced by 54% in low glucose with added ketone bodies treated cells compared to high glucose cells (**Figure 1E**).

### Ketogenic diet improved mitochondrial respiration and increased mitochondrial biogenesis

**Figure 2:**
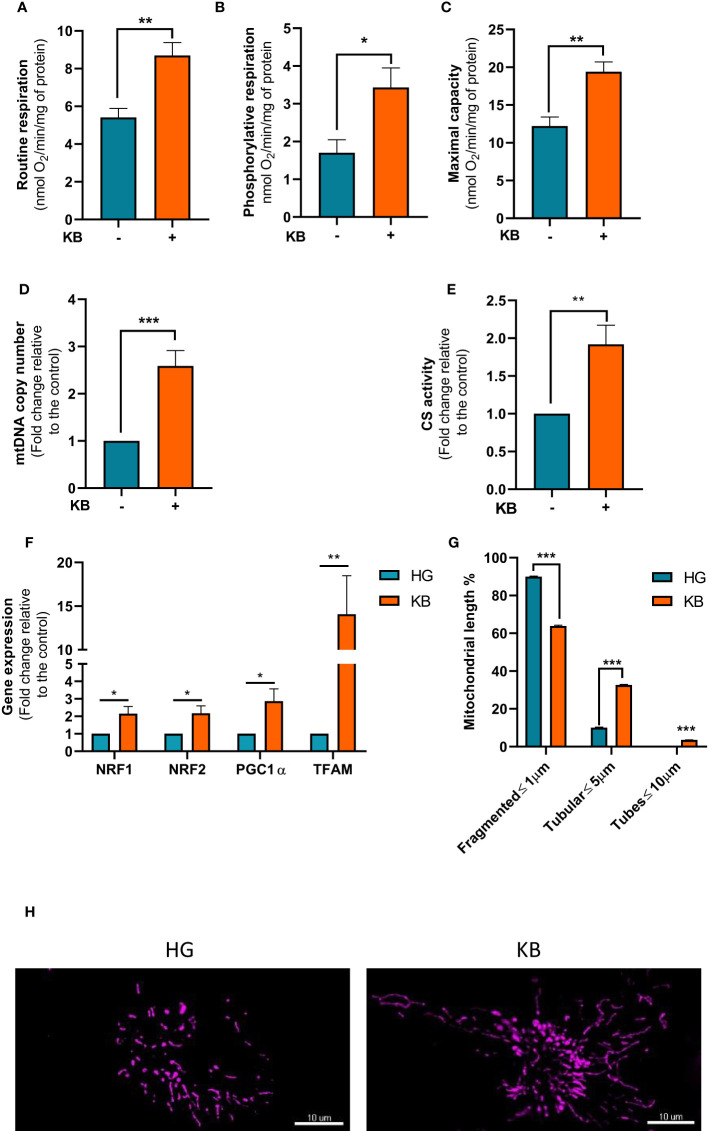
Mitochondrial respiration and metabolism in ACHN cells exposed to KB. HG = 25mM glucose, 2mM L-glutamine, 10% non-dialyzed FBS, 0mM ßHB, 0mM AcAc and KB = 2.8mM glucose, 2mM L-glutamine, 10% non-dialyzed FBS, 5mM ßHB, 5mM AcAc **(A)** Oxygraphic measurements of mitochondrial basal respiration (n=10). **(B)** Cellular phosphorylating respiration measured in nmol O2/min/mg of protein (n=10). **(C)** Maximal cellular oxidative capacity determined with FCCP titration (n=10). **(D)** Quantification of mtDNA copy number in ACHN cells exposed to KB compared to untreated cells (n=4). **(E)** Citrate synthase (CS) measurement (n=6). **(F)** Gene expression of genes involved in mitochondrial biogenesis using qPCR in ACHN cells (n=4). **(G)** Quantification of fragmented, tubular and tube mitochondrial lengths (HG n=38; KB n=44). **(H)** Representative images of the mitochondrial network of ACHN exposed (right panel) or not to KB (left panel). Data are presented as mean ± SEM and asterisks (*) indicates significant differences (p ≤ 0.05), (**) (p ≤ 0.001), (***) (p ≤ 0.0001).

To confirm our hypothesis, mitochondrial respiration in intact ACHN cells was measured. The basal respiration was significantly increased in ACHN by 1.6 fold change (61%) compared to untreated cells after 6 days of LG in addition to KB exposure (**Figure 2A**). Furthermore, the part of OXPHOS respiration used to produce ATP was significantly increased with a 2 fold change in cells grown in LG with added KB compared to HG cells (**Figure 2B**). However, caution is needed in extrapolating OXPHOS respiration to ATP synthesis through OXPHOS in tumor cells (26). Similarly, the maximal capacity of respiration was increased by 1.6 fold change (**Figure 2C**). In addition, LG with added KB exposure significantly increased mtDNA copy number in ACHN cells with a 2.6 fold change (159%) compared to untreated cells after 6 days of low glucose coupled with ketone bodies exposure, supporting the induction of mitochondrial biogenesis (**Figure 2D**). In parallel citrate synthase (CS) activity considered as a key marker of mitochondrial mass, was significantly increased by 98% in treated cells compared to control cells (**Figure 2E**).

The expression of genes involved in mitochondrial biogenesis including, NRF1, NRF2, PGC1a and TFAM were assessed in treated cells (**Figure 2F**). After 6 days of low glucose in addition to ketone bodies exposure gene expression of selected mitochondrial genes were significantly induced by NRF1 by 2.1, NRF2 by 2.2, PGC1a by 2.9, and TFAM by 14.1 fold changes strongly suggesting an increase of mitochondrial biogenesis. Moreover, LG with added KB significantly improved the mitochondrial network by reducing fragmented mitochondria by 29% and increasing mitochondrial lengths by 225% with the appearance of tubular structures demonstrating the beneficial effect of LG coupled with KB on mutant cells (**Figures 2G, H**).

### The *in vivo* ketogenic diet decreased tumor growth proliferation and increased gene expression of CD274 in a grafted mouse model

**Figure 3:**
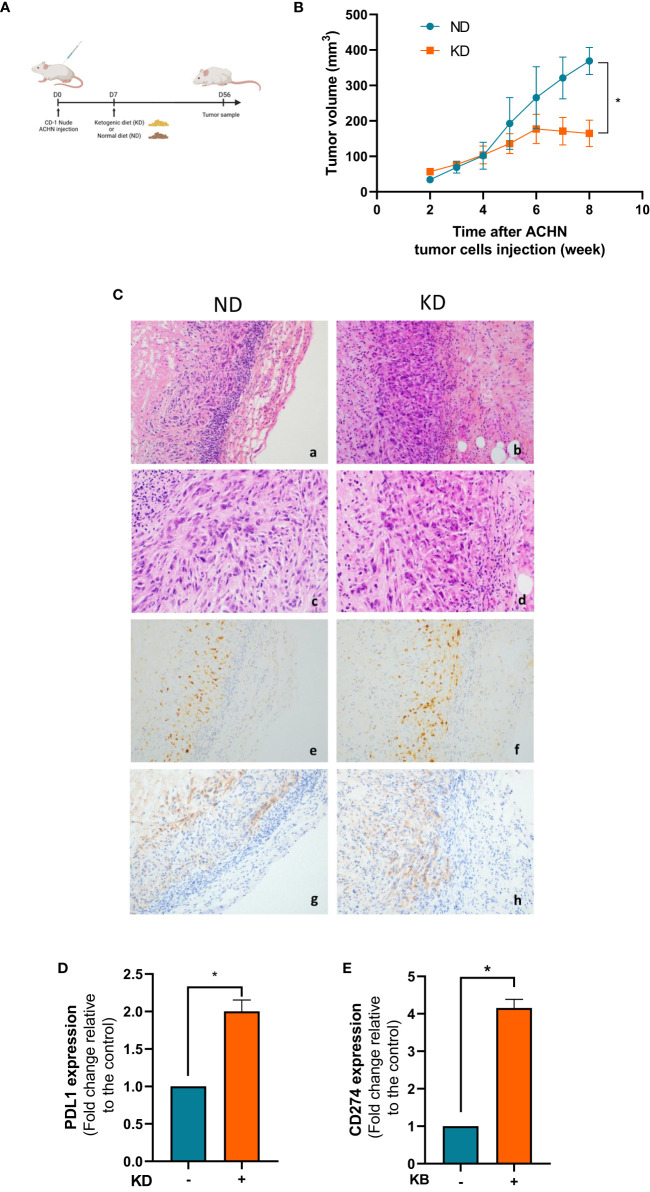
KD decreases tumor growth proliferation *in vivo* and increases gene expression of CD274. **(A)** Nude mice were grafted with ACHN cells at day 0 and treated with KD or not from day 7 to day 56. **(B)** Measurements of tumor volumes were assessed once a week during 8 weeks. **(C)** HES and immunohistochemical staining performed at day 56 to assess PD-L1 expression and Ki67 index in normal diet (upper panel) and ketogenic diet (downer panels) groups. Representative images are presented at objective x20 (a,b,g,h) x40 (c,d) and x10 (e,f). (a,b,c,d): HES staining showing undifferentiated high-grade tumor cells spindle-shaped with marked cytologic atypia. (e,f): Ki67 proliferative index (anti-mib1) was similar between the two groups regardless of the diet (g,h): PD-L1 expression was slightly increased in KD tumor cells. **(D)** Relative mRNA expression analyzed by RT-qPCR in tumor xenograft at day 56. **(E)** Relative mRNA expression analyzed by RT-qPCR in ACHN exposed or not to KB. Data are presented as mean ± SEM and asterisks (*) indicates significant differences (p ≤ 0.05).

We further analyzed the consequences of KD on the growth proliferation of ACHN cells grafted mice. Animals were subdivided into two groups, one group with a normal diet (ND) and one group with a 2:1 KD (**Figure 3A**). Tumor growth was regularly measured once a week in both mouse groups. The mean tumor growth was 930 ± 50% in the control group vs 190 ± 70% in the KD group after 8 weeks of KB exposure (p<0.001) (**Figure 3B**). The mean blood ketone level was 0.78 (± 0.16) mmol/L in the control group, and 1.12 (± 0.16) mmol/L in the ketogenic group (p=0.001) (**Supplementary Data**). The mean blood glucose level was 7.8mM in the control group and 6.8mM in the ketogenic group (p=0.01) (**Supplementary Data**). In mice, ketosis was quickly reached without weight change between both groups (**Supplementary Data**).

Since immunotherapies are now widely used in kidney cancer therapies, we looked at the influence of KD on the PD-1 immune-checkpoint inhibitor. In the KD treated mouse group, we confirmed the over-expression of PD-L1 by immunochemistry on the surface of tumor cells (**Figure 3C**). The Ki67 tumor proliferation rate was not significantly different between the two conditions (around 80%) (**Figure 3C**). Gene expression of CD274, which encodes the immune inhibitory receptor ligand of PD-L1 was increased by 2 fold change (99%) in mouse tumors in the KD group compared to ND group (**Figure 3D**). This increase was further confirmed by qPCR in ACHN cells *in vitro* revealing that PD-L1 was significantly 4 fold change increase after 6 days of exposed to low glucose environment in addition to ketone bodies (**Figure 3E**).

### The efficacy of immunotherapy treatment was improved by adjuvant ketogenic diet

**Figure 4:**
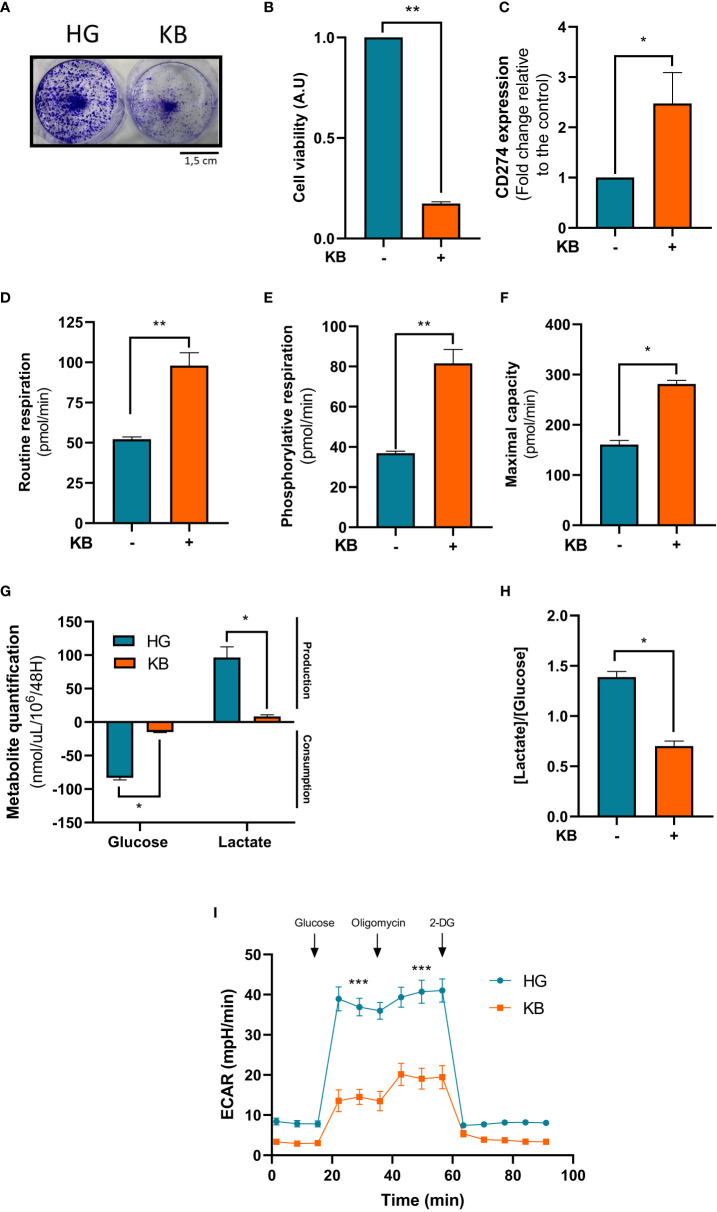
Renca cells exposed to KB overexpressed CD274 while reducing cell number and increasing mitochondrial respiration. HG = 25mM glucose, 2mM L-glutamine, 10% non-dialyzed FBS, 0mM ßHB, 0mM AcAc and KB = 2.8mM glucose, 2mM L-glutamine, 10% non-dialyzed FBS, 5mM ßHB, 5mM AcAc **(A, B)** Renca cell number was estimated by crystal violet after being exposed to KB compared to untreated cells (HG for high glucose media) for 6 days (n=6). **(C)** Relative mRNA CD274 expression was analyzed by RT-qPCR Renca cells treated or not with KB (n=6). **(D)** Oxygraphic measurements of mitochondrial routine respiration (n=6). **(E)** Cellular phosphorylating respiration measured in nmol O2/min/mg of protein (n=6). **(F)** Maximal cellular oxidative capacity of Renca cells (n=6). **(G)** Glucose consumption and lactate production were assessed in cell supernatant from untreated HG-Renca compared to KB-Renca (n=4). **(H)** Ratio of lactate/glucose was estimated in supernatants from HG-Renca and KB-Renca (n=4) **(I)** ECAR production measurements (n=6). (*) indicates significant differences (p≤0.05), (**) (p≤0.001), (***) (p≤0.0001).

Next we used Renca cells, a murine cell line of renal carcinoma exposed for 6 days with low glucose in addition to ketone bodies. Number of Renca cells was significantly lower in LG with added KB condition than in HG condition with a significant reduction of 83% viability as revealed by crystal violet staining (**Figures 4A, B**). Mitochondrial respiration rates of Renca cells exposed for 6 days to low glucose coupled with ketone bodies compared to HG cells were measured. The basal respiration was significant induced with a 1.9 fold change (88%), ATP-linked respiration with a 2.2 fold change (121%) and the maximal respiration capacity was significantly increased in low glucose, KB treated cells than in HG (**Figures 4C–E, G**) confirming the significant improvement of oxidative mitochondrial metabolism already seen with the ACHN LG with added KB treated cells. Glucose consumption was significantly reduced by 83% in Renca cells under low glucose, KB treatment, also associated with a significant reduction of 91% of lactate production (**Figure 4G**). The reduction of lactate was confirmed with 54% decrease of the [lactate]/[glucose] ratio (**Figure 4H**). The level of acidification of Renca cells was significantly reduced by 63% in low glucose coupled with KB treated cells (**Figure 4I**).

**Figure 5:**
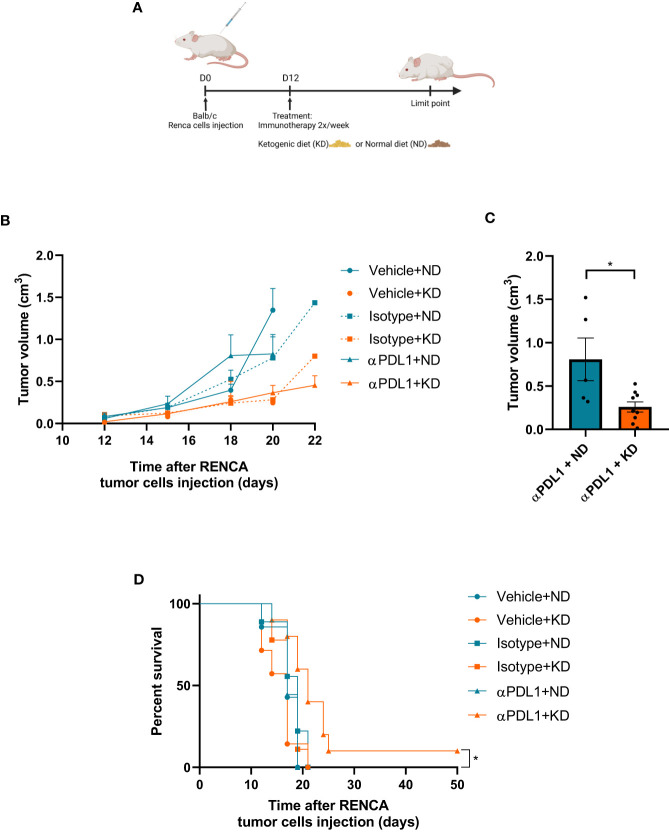
The efficacy of immunotherapy treatment is improved by adjuvant KD. **(A)** Balb/c mice were grafted with Renca cells at day 0 and treated with KD or not from day 12 to the end of the experiment. **(B)** Measurements of tumor volumes were assessed three times a week during 4 weeks for each group (10 mice per groups) exposed or not to KD and receiving anti-PDL1 mAb (αPDL1+ND or αPDL1+KD groups), isotype control (isotype+ND and isotype+KD groups) and PBS controls (vehicle+ND and vehicle+KD groups). **(C)** Comparison of tumor volume at day 18 between groups treated by anti-PDL1 and exposed to KD or ND. **(D)** Survival of mice exposed to KD or ND, treated with anti-PDL1 mAb or isotype control or PBS (vehicle) after grafting of Renca cells. **(C)** Statistical significance was determined using Mann-Whitney U test. Data are presented as mean ± SEM. **(D)** Survival curves were done using Kaplan-Meier method and compared using the Log-rank test. *P<0.05.

To confirm the effect of KB on tumoral cells, syngeneic mice were treated with KD and/or anti-PD-L1 immunotherapy 12 days (**Figures 5A**) after been grafted with Renca cells. Tumors from mice under KD treated or not presented a reduced volume (**Figure 5A**). Eighteen days after tumor graft, mice under KD and treated with anti-PDL1 mAb presented a significantly reduced tumor volume compared to mice under KD treated with isotype control (**Figures 5B, C**). Interestingly, treatment with anti-PDL1 mAb combined with KD was significantly efficient in one mouse under KD treated with anti-PDL1 mAb which survived while all mice from the other groups died. (**Figure 5D**).

## Discussion

In this study, we observed that *in vitro* KB exposure led to a significant reduction of proliferation of ACHN and Renca cells. In ACHN cells, the overall increase in the respiration rates was also combined with a significant increase of mitochondrial mass and biogenesis favoring a metabolic switch towards oxidative phosphorylation. These observations were in line with previous studies showing that KB induces a decrease in tumor cell growth in renal clear cell carcinoma (27). We attempted to replicate the experiments by culturing Renca and ACHN cells in a glucose-deficient medium without ketone bodies to obtain additional control. This manipulation was challenging to develop due to the lack of cell proliferation, despite regular changes in the culture medium (data not shown). We acknowledge the limitations of our study, as we cannot entirely rule out the possibility that the observed *in vitro* changes may stem from low glucose levels rather than the presence of ketone bodies. Additional control experiments would be crucial to ascertain whether the observed effects result from restricted glucose levels or limited ketone levels.

We confirmed on a renal tumor cell xenograft in nude CD-1 mice that KD was associated with a significant decrease of tumor growth *in vivo*. These results are different from Liśkiewicz AD et al. who described an opposite effect of the KD promoting tumor growth in an Eker rat model carrying *TSC2* mutation. This *in vivo* model was different from ours by several points. The KD regimen was much more restrictive with an 8:1 ratio vs 2:1 ratio in our study. It was also a non-malignant renal tumor model carrying the TSC2 mutation which is associated with tuberous sclerosis of Bourneville and with renal angiomyolipomas, a completely different benign histological and genetic model of renal tumor compared to renal clear cell carcinoma (28). This decrease of ACHN tumor growth *in vivo* was in line with our *in vitro* results showing that the growth proliferation of ACHN cells exposed to KB in culture was significantly reduced and associated with improved mitochondrial metabolism.

After 8 weeks of KD, tumors grafted in CD1 nude mice over-expressed PD-L1 transcript. This protein is involved in immune checkpoint programmed death-1(PD1)/PD-L1 interaction and constitutes a new target for treatments in the metastatic renal cancer management. Current pivotal phase 3 studies showed that PD-1 ICI-based combinations were more efficient than the VEGFR-TKI Sunitinib, a previous standard of care, leading to the approval of four new regimens as guideline-recommended first-line treatments (29). The overexpression of PD-L1 on the surface of tumor cells was considered as a positive marker of response to metastatic renal cancer treatment (7). PD-L1 positivity of the tumor was associated with an improved objective response rate and a prolonged progression free survival in mRCC patients receiving ICI treatment (30). These observations were confirmed in our immunocompetent *in vivo* model of renal carcinoma (Renca). This would suggest that KD may result in slower growth of a renal tumor model and modify the intra-tumoral immunity as previously shown by Ferrere et al. (31).The PD-L1 protein is mainly known to have an inhibitory role on cytotoxic CD8 T lymphocytes (CTL) and its inactivation by mAb treatment can restore specific CTL efficacy. Moreover, in our *in vivo* model as previously described, cancer cell metabolism modifications by KD enhanced critical key metabolite pathways such as fatty acid and glutamine pathways which are involved in PD-L1 expression increase (32). In our immunocompetent *in vivo* model of renal carcinoma (Renca), we noted minimal impact of anti-PD1 monotherapy alone on primary tumor growth, consistent with clinical observations where only 6% of patients exhibit a notable decrease in primary renal tumor size under nivolumab treatment (33). The experiment was conducted two times, each instance involving a mouse displaying an extended response to the treatment. However, the dataset lacks statistical adequacy to draw conclusions regarding the long-term complete response rate. Regrettably, due to ethical constraints pertaining to animal welfare, further iterations of the manipulations were precluded. Nevertheless, these findings demonstrate a similarity to clinical observations, particularly in the context of first-line immunotherapy for metastatic kidney cancer, where the long responder rate typically fluctuates by around 20% (34).

Tumor cells exposed *in vivo* to KB due to KD reduced their lactate production. This reduction of lactate in the cellular environment could impact the tumor environment by changing the polarization of macrophages modifying the tumor associated macrophages into macrophages with anti-tumor features.. We showed that switching metabolism towards OXPHOS causes a decrease in lactate production and therefore would reduce acidosis of the extracellular medium and then polarize cells into anti-tumor M1 macrophages (35). The ketogenic diet, while lowering tumor growth, would then promote the immune response. The lactate-induced acidosis in the microenvironment suppresses the immune response. A reduction in lactic acidosis could induce a positive signal towards increased expression of PDL1 within renal tumor cells but also maybe potentiate the therapeutic role of immunotherapies. We also reported the reduction of tumor growth proliferation from primary ccRCC from renal cell patients (**Supplementary Files**). Interestingly, the most aggressive tumor cells from ISUP 4 RCC displayed a reduction of *in vitro* growth proliferation in the presence of KB.

These initial *in vitro* and *in vivo* data suggest that KD may be of benefit in combination with anti-PDL1 treatments in patients with metastatic RCC. Our first pre-clinical results led to the establishment of a pilot study (CETOREIN study, NCT04316520) aiming to evaluate the tolerance of KD associated with standard of care in patients with metastatic RCC. Furthermore, other research groups are investigating the impact of the ketogenic diet on renal cancer. The KETOREIN trial (NCT05119010), conducted by the Gustave Roussy Institute, aims to assess the efficacy of the ketogenic diet in patients receiving first-line treatment with nivolumab and ipilimumab for metastatic renal cancer. The initial clinical data from these two studies could confirm the feasibility and potential benefits of the ketogenic diet in patients with kidney cancer. However, until results are available, preclinical study data should be interpreted cautiously, and this dietary regimen should only be utilized for patient with kidney cancer within the context of clinical trials under medical and dietary supervision.

## Conclusion

KD significantly reduced renal tumor cell growth proliferation. It also slowed down the tumor growth of ACHN xenografts in CD1 nude mice. We finally observed that a change in intratumoral immunity was associated with PD-L1 overexpression on the surface of tumor cells induced by KD. This dual effect of KD may be used as an adjuvant therapy in synergy with current first line immunotherapies in the management of patients with metastatic renal cancer. Further studies are needed to confirm the therapeutic benefit of KD as an adjuvant regimen for immunotherapies.

## Data availability statement

The datasets presented in this study can be found in online repositories. The names of the repository/repositories and accession number(s) can be found in the article/**Supplementary Material**.

## Ethics statement

All patients were from the national database “UroCCR” (NCT03293563, CNIL authorization number: DR-2013-206) and were informed about the purpose of the study, and their written consent was obtained. The studies were conducted in accordance with the local legislation and institutional requirements. The participants provided their written informed consent to participate in this study. Animal studies were performed in strict agreement with the French ethic legislation, approved by the local ethic committee for animal experimentation (CEEA des Pays de la Loire) and the French ministry of research (APAFIS#6787-2016092008289183v1 and APAFIS#23580-20200121317125532v4). The study was conducted in accordance with the local legislation and institutional requirements. Written informed consent was obtained from the individual(s) for the publication of any potentially identifiable images or data included in this article.

## Author contributions

JR: Investigation, Writing – original draft. CB: Conceptualization, Formal analysis, Supervision, Validation, Writing – review & editing. MBe: Investigation, Writing – original draft. MBa: Conceptualization, Writing – review & editing. CA: Investigation, Writing – original draft. CR: Writing – review & editing. SB: Investigation, Writing – original draft. JB: Project administration, Supervision, Writing – review & editing. MF: Investigation, Writing – original draft. SL: Writing – review & editing. AC: Software, Writing – review & editing. DH: Project administration, Supervision, Writing – review & editing. VP: Conceptualization, Methodology, Project administration, Supervision, Validation, Writing – review & editing, Writing – original draft. PB: Conceptualization, Formal analysis, Funding acquisition, Investigation, Methodology, Project administration, Resources, Supervision, Validation, Visualization, Writing – original draft, Writing – review & editing.
