# Supplementary material for: Ketogenic diet enhances the anti-cancer effects of PD-L1 blockade in renal cell carcinoma
Source: Front Endocrinol (Lausanne). 2024 May 17;15:1344891. doi: 10.3389/fendo.2024.1344891 (PMC11154604; doi:10.3389/fendo.2024.1344891)
Supplement: Supplementary file 1 [file DataSheet_1.pdf]

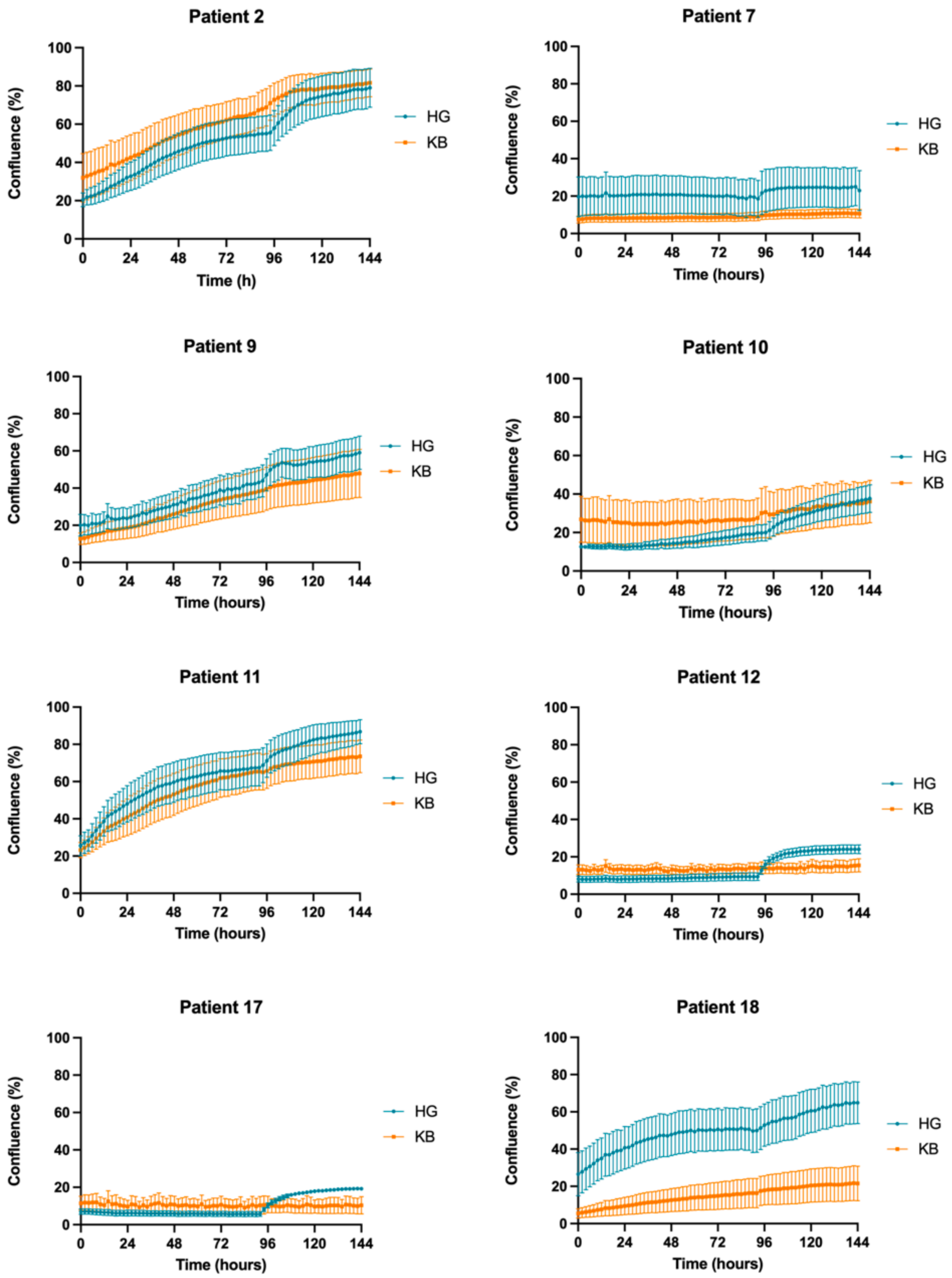

Supplemental figure 1. Ketone bodies reduced growth cell proliferation of primary kidney cancer cells

Supplemental table 1 : Characteristics of the five patients from whom the primary cultures were obtained. T stage refers to the American Joint Committee on Cancer (AJCC) “Tumor Node Metastasis” (TNM) classification (1). T describes the size of the original (primary) tumor. N describes lymph nodes that are invaded. M describes distant metastasis. ISUP refers to the International Society of Urological Pathology score

	Age (year)	Tumor size cm	ISUP	TNM
**Patient 2**	54	4.5	2	pT1b N0 M0
**Patient 7**	51	8.5	2	pT2a N0 M0
**Patient 9**	58	7	2	pT1b N0 M0
**Patient 10**	61	11.5	3	pT3a N0 M1
**Patient 11**	65	8	4	pT3a N0 M0
**Patient 12**	84	8	3	pT2a N0 M0
**Patient 17**	73	13	4	pT3a N0 M0
**Patient 18**	64	9.5	4	pT3a N0 M0

Primary cell isolation and culture

Once the nephrectomy was performed, the surgical specimen was transported in a fresh buffered salt solution to the anatomopathological department for macroscopic examination. After anatomopathological analysis, a 1 cm3 non-necrotic fragment of the tumor was selected and stored at 4 °C in 50 mL of PBS (phosphate-buffered saline, Sigma Aldrich, St. Louis, MO, USA) until its use. Following this, mechanical digestion was performed under sterile conditions using two cold scalpels. The resulting millimeter-sized pieces were incubated at 37 °C for 90 min under agitation in the presence of DMEM 4.5 g/L glucose, 1% pyruvate, 1% penicillin–streptomycin (Sigma Aldrich), 0.2 Wünsch units/mL liberase (Roche, Basel, Switzerland) and 0.1 mg/mL DNase (Roche). The solution obtained was sieved through a 70 µm filter (SPL Life Sciences, Pocheon, Republic of Korea). Suspended cells were pelleted for 5 min at 300× g. Next, cells were subcultured in culture medium 1 containing 33% of Mix AmnioMAX™ (AmnioMAX™ medium + AmnioMAX™ supplement) (Thermo Fisher Scientific, Waltham, MA, USA), 10% of Fetal Bovine Serum (FBS) (Thermo Fisher Scientific), 1% of RGEM1™ (LONZA, Basel, Switzerland), 1% of Glutamine (Sigma-Aldrich), 1% of penicillin and streptomycin (Sigma-Aldrich) and 54% of DMEM 4.5% of glucose (Sigma-Aldrich) prewarmed to 37 °C and transferred to 25 cm2 culture flasks (SPL Life Sciences). After overnight incubation (37 °C, 5% CO2), culture medium was renewed, and the unattached cells were removed. This procedure was repeated every 24 h until the flasks were free of debris and unattached cells. Thereafter, the culture medium was changed every 2 to 3 days until confluence. At 70–80% confluence, cells were washed with PBS and detached using trypsin (20 µL/cm2 trypsin-EDTA 1X (trypsin 0.05%; EDTA 0.02%), Sigma-Aldrich) for 5 min. Cells were then pelleted by 5 min centrifugation at 500× g and resuspended in 2 mL of culture medium 2 containing 33% of Mix AmnioMAX™ (Thermo Fisher Scientific), 10% of FBS (Thermo Fisher Scientific), 1% of Glutamine (Sigma-Aldrich), 56% of DMEM and 4.5% of glucose (Sigma-Aldrich). Cell count was performed by trypan blue exclusion and Neubauer hemocytometer. Some of the cells were frozen at −80 °C, and the rest were transferred to a 75 cm2 flask with culture medium 2. Once 80% confluence was obtained, the cells were transferred to a T175 cm2 flask for amplification. Culture medium 1 was optimized for survival

after mechanical and chemical digestion and to avoid culture infections during tissue handling. It contained growth factor supplements for use with renal epithelial cell basal medium (1% REGM1™ (Renal Epithelial Cell Growth Medium SingleQuots™ Kit, LONZA, Bâle, Switzerland)) and 1% antibiotic (penicillin and streptomycin, Sigma-Aldrich). After the first passage, these elements were no longer needed and could constitute an avoidable bias for cell growth.

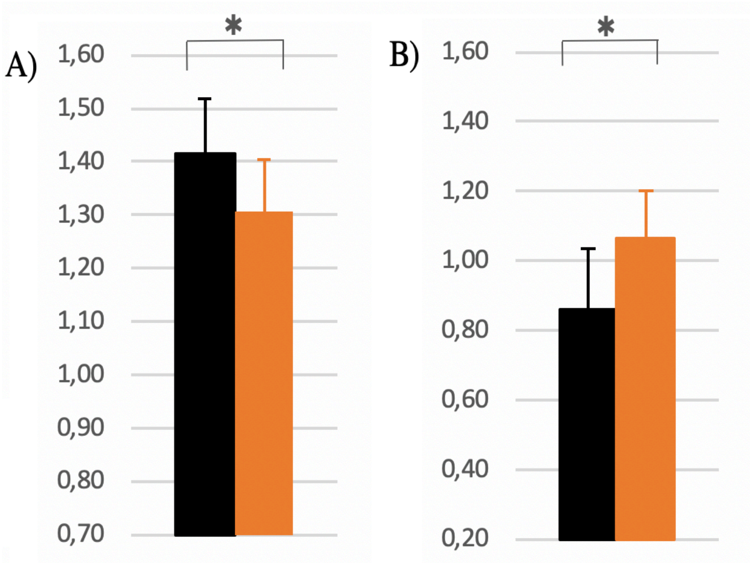

Supplementary figure 2: Comparison of blood glucose (A) and ketone levels (B) of CD1 mice on a standard (black) or ketogenic 2.1 (orange) diet. *p<0.05

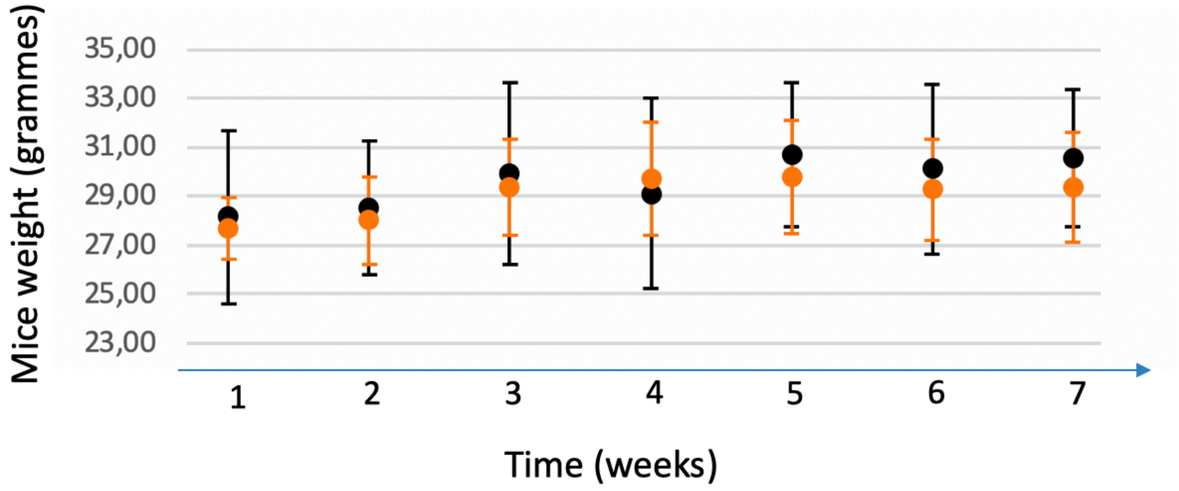

Supplementary figure 3: Evolution of the weight of the CD-1 mice (grammes) over time (weeks) and according to a classic diet (black) or a ketogenic diet 2.1 (orange)
